# Outcomes and resource use in Brazilian ICUs: results from the ORCHESTRA study

**DOI:** 10.1186/cc14585

**Published:** 2015-03-16

**Authors:** M Soares, DC Angus, JI Salluh, AB Cavalcanti, F Colombari, R Costa, E Silva, A Japiassu, JM Kahn, FA Bozza

**Affiliations:** 1 DOr Institute for Research and Education - IDOR, Rio De Janeiro, Brazil; 2 University of Pittsburgh Medical Center, Pittsburgh, PA, USA; 3 Hospital do Coracao, São Paulo, Brazil; 4 Hospital Alemao Oswaldo Cruz, São Paulo, Brazil; 5 Hospital Quinta DOr, Rio de Janeiro, Brazil; 6 Hospital Israelita Albert Einstein, São Paulo, Brazil; 7 Rede Amil de Hospitais, Rio de Janeiro, Brazil

## Introduction

The aim was to evaluate outcomes and resource use and to investigate the association between organizational factors and efficient resource use in a large sample of Brazilian ICUs.

## Methods

A retrospective cohort study in 59,483 patients (medical admissions: 39,734 (67%)) admitted to 78 ICUs (private hospitals, *n *= 67 (86%); medical or medical-surgical, *n *= 62 (79%)) during 2013. We retrieved demographic, clinical and outcome data from an electronic ICU quality registry (Epimed Monitor System). We surveyed ICUs using a standardized questionnaire regarding hospital and ICU structure, organization, staffing patterns, process of care and family care policies. Efficient resource use was assessed by estimating standardized mortality rates (SMR) and standardized resource use (SRU) adjusted for the severity of illness according to the SAPS 3 score, as proposed by Rothen and colleagues [1].

## Results

The median admissions per center was 898 (IQR 585 to 1,715) and SAPS 3 score was 41 (33 to 52) points. Median ICU length of stay was 2 (1 to 5) days, and ICU and hospital mortality rates were 9.6% and 14.3%, respectively. Estimated SMR and SRU were 0.97 (0.72 to 1.15) and 1.06 (0.89 to 1.37), respectively. There were 28 (36%) most efficient (ICUs with both SMR and SRU <median), 28 (36%) least efficient (ICUs with both SMR and SRU >median), 11 (14%) overachieving (ICUs with low SMR and high SRU) and 11 (14%) underachieving (ICUs with high SMR and low SRU) ICUs. Most efficient ICUs were usually located in private and accredited hospitals, with step-down units and training programs in critical care. In univariate analyses comparing most and least efficient ICUs, ≥2 clinical protocols (OR = 7.22 (95% CI, 1.41 to 36.97)) and graduated nurse/bed ratio >0.25 (OR = 4.40 (1.04 to 18.60)) were associated with efficient resource use. Daily checklists also tended to be associated with efficient resource use (OR = 2.89 (0.95 to 8.72), *P *= 0.057).

## Conclusion

We observed a great variability in outcome and resources in a large sample of Brazilian ICUs. Implementation of clinical protocols and nursing staffing patterns can be targets to improve the efficiency in resource use in emerging countries such as Brazil.

## Acknowledgements

Funded by IDOR, CNPq and FAPERJ. Endorsed by BRICNet.
